# Response to toripalimab combined with radiotherapy in advanced non-small cell lung cancer-not otherwise specified: A case report

**DOI:** 10.1097/MD.0000000000027581

**Published:** 2021-10-22

**Authors:** Yonglong Jin, Wenjing Xiao, Xintong Wang, Yinshi Cui, Bo Li, Xiguang Liu

**Affiliations:** a Department of Radiotherapy, Affiliated Hospital of Qingdao University, Qingdao, China; b Department of Cardiology, Qingdao Fuwai Cardiovascular Hospital, Qingdao, China.

**Keywords:** immune checkpoint inhibitors, non-small cell lung cancer-not otherwise specified, radiotherapy, remote effects

## Abstract

**Rationale:**

The targeting of signal transduction through programmed cell death receptor-1 (PD-1) and its ligand programmed cell death-ligand 1 (PD-L1) in patients with non-small cell lung cancer (NSCLC) has been widely applied in clinical research. However, the subtypes and treatment patterns that predict responses to PD-1/PD-L1 inhibitors are not fully understood. Biomarkers, such as PD-L1 expression, tumor mutation load, and DNA mismatch repair defects, have been used to screen patients who respond to PD-1/PD-L1 inhibitors, but the appropriate treatment mode requires further investigation. Immune checkpoint inhibitors combined with radiotherapy provide benefits from remote effects, especially in NSCLC patients with increased PD-L1 expression.

**Patient concerns:**

We report a 64-year-old man who presented with left back pain for 40 days. A computed tomography scan showed a mass in the right upper lobe of the lung, with metastases in the right hilar and mediastinal lymph nodes.

**Diagnosis:**

NSCLC-not otherwise specified was diagnosed by computed tomography-guided lung biopsy.

**Interventions:**

After the failure of first-line chemotherapy, next-generation sequencing was performed for comprehensive gene analysis, and PD-L1 expression levels were evaluated by immunohistochemistry. The patient was treated with toripalimab (a PD-1 inhibitor) concurrently with radiotherapy for bone metastases.

**Outcomes:**

The detection results showed a high tumor mutation burden and increased PD-L1 expression. On the basis of these findings, the patient received toripalimab (PD-1 inhibitor) combined with radiotherapy for bone metastases. Partial response was achieved after 3 cycles, and the patient showed stable disease at the end of the sixth and ninth cycles of toripalimab. The patient was followed up for 26 months. At present, the patient is receiving toripalimab maintenance treatment, which has been well-tolerated without adverse events.

**Lesson:**

Toripalimab combined with radiotherapy may exert a synergistic anti-tumor effect through remote effects in advanced or metastatic NSCLC with high PD-L1 expression. However, the specific treatment mode requires further confirmation by the investigation of additional cases.

## 1 Introduction

Lung cancer is the leading cause of cancer deaths worldwide, and non-small cell lung cancer (NSCLC) represents approximately 80% of the cases. The survival rate of advanced NSCLC is poor, with a global 5-year survival rate below 20%.^[1]^ Currently, immune checkpoint inhibitors, including programmed cell death receptor-1 (PD-1) and programmed cell death-ligand 1 (PD-L1), have evolved as the most hopeful and attractive therapeutic modalities for NSCLC.^[2]^ In this article, we describe a patient with NSCLC who showed a durable response to toripalimab (PD-1 inhibitor) combined with radiotherapy after failure of first-line chemotherapy.

## 2 Case report

A 64-year-old man was admitted to our hospital on December 28, 2018 owing to left back pain for 40 days. The pain degree was evaluated by using the Numerical Rating Scale (NRS). The NRS score of the patient was 7. The patient on physical examination, a mass of approximately 15 × 10 cm was seen on the left back. No superficial lymphadenopathy or other remarkable findings were noted, except for coarse breathing sound over the right upper lung area and a certain tenderness over the left back and left sacrum. A computed tomography (CT) scan showed an approximately 6.2 × 3.5 cm mass in the right upper lung lobe, with metastases in the right hilar and mediastinal lymph nodes. The bilateral posterior chest wall was involved. Destruction in the T8 and T9 thoracic spine bone was observed. A puncture biopsy in the right upper lobe under CT guidance provided a diagnosis of poorly differentiated carcinoma that was positive on subsequent bronchoscopy brushing cytology. A biopsy from an occlusive lesion in the left upper lobe bronchus led to a diagnosis of poorly differentiated carcinoma positive for CK7 and negative for TTF-1, p40, p63, CD56, CK5/6, and Napsin-A immunostaining, favoring a diagnosis of NSCLC-not otherwise specified with cT4N2M1 stage IV.

**Figure 1:**
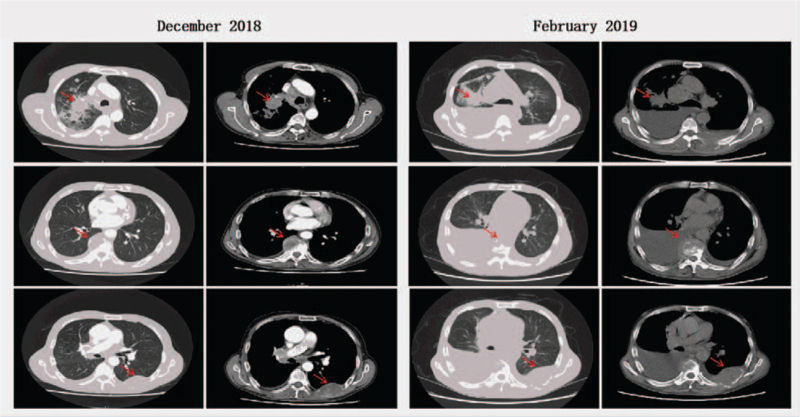
CT scans of the patient. The primary lesion and metastatic lymph nodes in the right lung had not shrunk, and the right pleural effusion had increased in size on the February 2019 scan compared with the previous scan. The curative effect was evaluated as disease progression. CT = computed tomography.

From January 12, 2019 to February 12, 2019, a second cycle of paclitaxel liposomes (175 mg/m^2^) and nedaplatin (120 mg/m^2^) of systemic chemotherapy and pain management was conducted. The cycle of chemotherapy is 21 days. After 2 cycles of chemotherapy, chest CT showed that the primary focus and metastatic lymph nodes in the right lung had not significantly shrunk, the right pleural effusion had increased, and the pain intensity was increased with an NRS score of 8. The curative effect was evaluated as progressive disease according to the Response Evaluation Criteria in Solid Tumors Version 1.1 (RECIST 1.1) (Fig. 1). On February 26, 2019, puncturing and drainage of the right pleural effusion was performed, and a total of 1500 mL of bloody pleural effusion was drained. Pathological examination of the pleural effusion cells revealed malignant tumor cells, and subsequent gene analysis indicated epidermal growth factor receptor/anaplastic lymphoma kinase-negative (3D Med/NGS), PD-L1-positive rate of 50% (Ventana/sp263 Kit), tumor mutation burden 27.42 muts/mb (3D Med/NGS), and microsatellite stable (3D Med/NGS).

**Figure 2:**
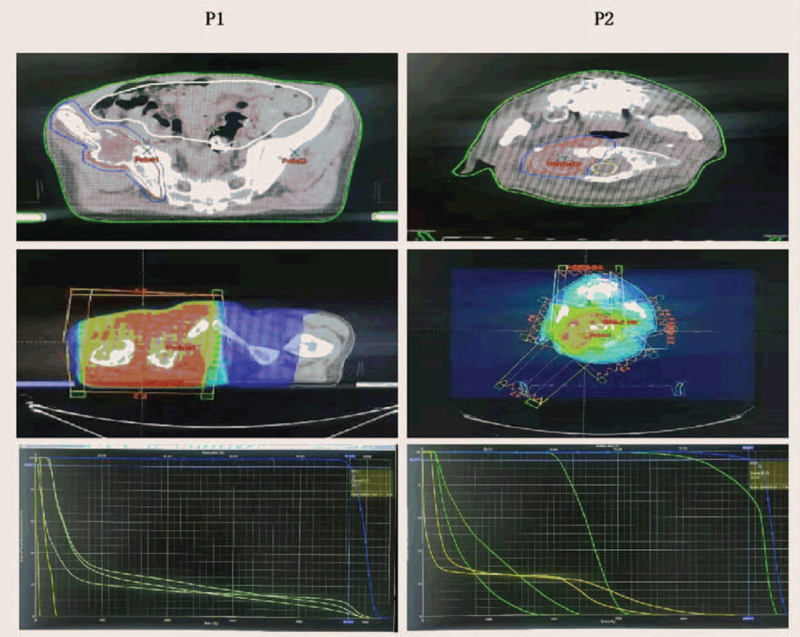
External radiotherapy for bone metastases from April 2019 to May 2019. The specific radiotherapy regimen was as follows: 6 mV X-ray, planning target volume covered by the 95% isodose curve, DT 5000 cGy/25 f/5 w (P1, right iliac bone), and DT 5000 cGy/25 f/5 w (P2, skull base and first cervical vertebra).

**Figure 3:**
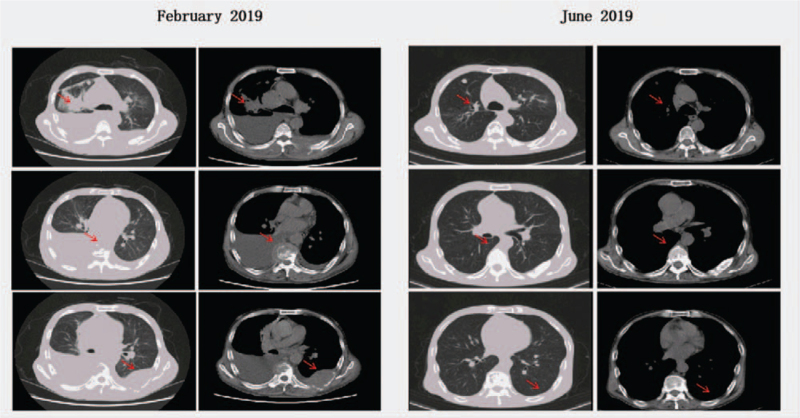
After the third cycle of PD-1 inhibitor treatment and local radiotherapy, the primary lesion in the right lung, metastatic foci in the posterior chest wall, and right hilar and mediastinal lymph nodes were markedly reduced, and the pain was considerably relieved. Combined with the physical examination and auxiliary examination findings, the curative effect was evaluated as partial remission. PD-1 = programmed cell death receptor-1.

After multidisciplinary case discussion, toripalimab (240 mg on day 1 of each 3-week cycle) was administered with oxycodone hydrochloride from March 3, 2019. During the treatment, external beam radiation therapy for a painful bone metastasis was carried out from April 4, 2019 to May 10, 2019. The radiotherapy schedule was 6-mV X-ray treatment, planning target volume covered by 95% isodose curve, DT 5000 cGy/25 f/5 w (P1, right ilium metastatic tumor), and DT 5000 cGy/25 f/5 w (P2, basis cranii, first cervical vertebra metastatic tumor) (Fig. 2). After the third cycle of toripalimab and bone metastasis focus radiotherapy, the sizes of the right lung primary lesion, posterior chest wall metastasis, and right hilar and mediastinal lymph nodes were all obviously reduced, the pain intensity was significantly mitigated with an NRS score of 4, and the X-ray showed similar multiple patchy shadows in the superior lobe of the 2 lungs as before (Fig. 3). Combined with the physical examination and auxiliary examination findings, the therapeutic effect evaluation was defined as partial response according to the RECIST 1.1.

**Figure 4:**
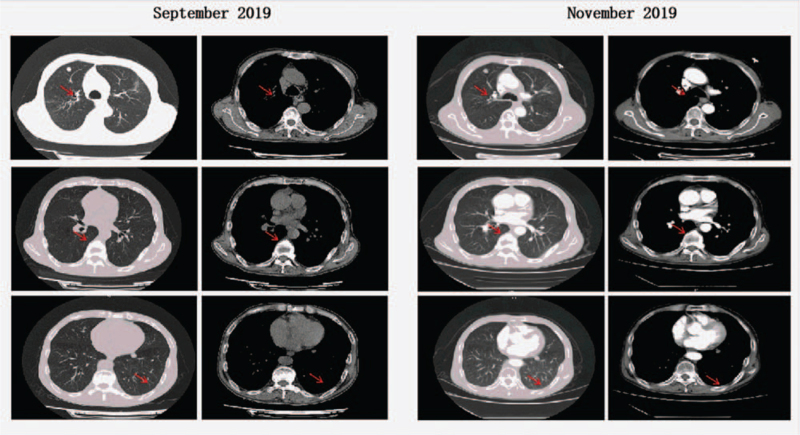
After the sixth and ninth cycles of PD-1 inhibitor treatment, the residual tumors in the right upper lobe and left posterior chest wall remained stable. The treatment effect was evaluated as stable disease. PD-1 = programmed cell death receptor-1.

**Figure 5:**
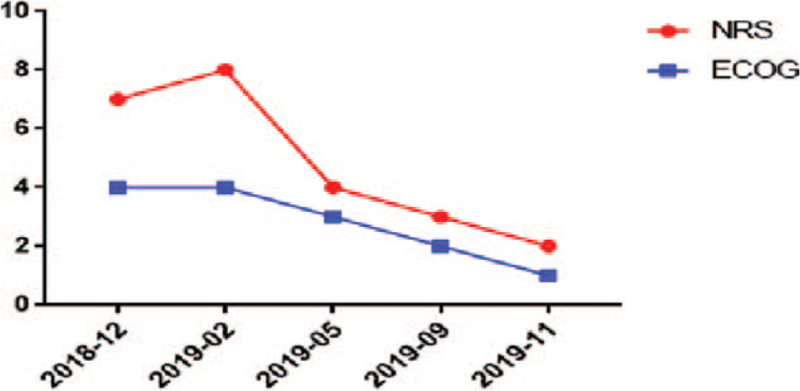
ECOG and NRS dynamic changes. ECOG scores: 0, ability for activities is completely normal; 1, can walk freely and engage in light physical activities, but cannot engage in heavy physical activities; 2, can walk freely, take care of themselves, and exercise for at least half of the day, but has lost the ability to work; 3, capable of limited self-care and stays in a bed or wheelchair for more than half of the day; 4, bedridden; and 5, death. NRS score: 0, pain-free; 1 to 3, mild pain; 4 to 7, moderate pain; 8 to 9, severe pain; and 10, most intense pain. ECOG = eastern cooperative oncology group, NRS = Numerical Rating Scale.

Afterward, the PD-1 inhibitor therapy was continued using the same methods described above. Meanwhile, the dosage of oxycodone hydrochloride prolonged-release tablets was gradually tapered as follows: 40 mg q12h by oral administration → 20 mg q12h by oral administration → 10 mg q12h by oral administration → 0 mg. At the end of the sixth and ninth cycles of toripalimab, the patient received a periodic review. The results demonstrated that the primary lesion in the right lung and the lymph node metastases were stable without new metastases, and that residual lesions were present in the superior lobe of the right lung and the left posterior chest wall. The therapeutic effect evaluation was rated as stable disease according to the RECIST 1.1 (Figs. 4 and 5).

**Figure 6:**
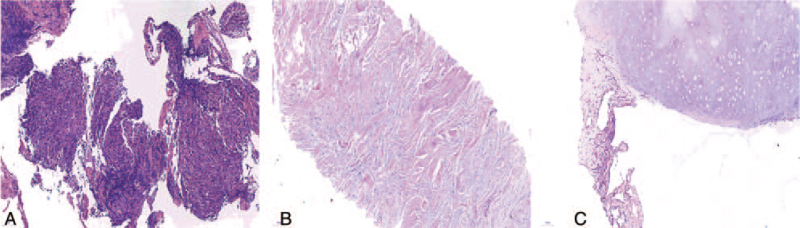
HE staining results for biopsy tissue taken under CT guidance. (A) Poorly differentiated non-small cell carcinoma (primary focus in the right upper lobe) (original magnification 100×). (B) Bone cells and a small number of inflammatory cells had infiltrated the left posterior chest wall tumor, but no malignant tumor cells were found (original magnification 100×). (C) A small amount of inflammatory cell infiltration, but no malignant tumor cells were found (original magnification 100×). CT = computed tomography.

To investigate the potential mechanism for the patient's inhibitor resistance and determine the follow-up therapeutic strategy, paracentesis was separately performed on the lesion in the left posterior chest wall under ultrasonic guidance and the residual lesion in the superior lobe of the right lung under CT guidance on August 16, 2019 and November 6, 2019, respectively, and the biopsy results were all negative (Fig. 6). The patient was followed up with duration of 26 months. At present, the patient is continuing to receive toripalimab maintenance treatment with good tolerance and stable disease, and no toxic or side effects related to the therapy have been identified.

## 3 Discussion

Generally speaking, approximately 75% of NSCLC patients are in the middle or advanced stage at diagnosis, and thus chemoradiotherapy has served as the traditional therapeutic method. However, its curative effect is by no means satisfactory, with a 5-year survival rate of less than 20%.^[3]^ Platinum-based doublet chemotherapy acts as the first-line treatment for advanced NSCLC patients without driver gene mutations; it has an objective remission rate of 25% to 35%, a median overall survival of 8 to 10 months, and a median progression-free survival of 4 to 6 months.^[4]^ For NSCLC patients with driver gene mutations, targeted therapy has shown obvious effects in terms of prolonged lifetime and progression-free survival.^[5]^ However, subsequent drug resistance remains inevitable. It is thus imperative to explore new therapeutic strategies for middle and advanced stage NSCLC patients who are unable to undergo surgery.

Tumor immunotherapy mainly employs the principles and methods of immunology to enhance cancer cell immunogenicity and sensitivity, activate and increase anti-tumor immune responses, and inject immune cells and effector molecules into the host to coordinate with the immune system in killing cancer cells and suppressing their growth.^[6]^ Immune checkpoints are important inhibition pathways in the immune system that are capable of suppressing excessive immune system activation to avoid harm to the physiological function of normal tissues.^[7]^ As research becomes more extensive, increasing numbers of immune checkpoints have been discovered, including PD-1/PD-L1, cytotoxic T-lymphocyte-associated-4, lymphocyte-activation gene 3, and killer inhibitory receptor. Among the immune checkpoints, PD-1/PD-L1 has gained the most attention from researchers. PD-1, which was first discovered by a Japanese scientist, is a type I transmembrane protein encoded by human programmed cell death protein 1 that is primarily expressed on the surface of activated T cells, B cells, and natural killer cells. Mechanically, PD-1 inhibitors block signal transduction between the immunosuppressive molecule PD-L1 on the surface of tumor cells and the receptor PD-1 on the surface of immune cells by competitively combining with PD-L1, thereby reactivating T cells and their immune monitoring effects.^[8]^ In 2015, the Food and Drug Administration approved the first immune checkpoint inhibitor, nivolumab, as a therapy for patients with advanced NSCLC, meaning that immunological therapy could become a new type of therapeutic method after chemoradiotherapy and targeted therapy.^[9]^

A series of domestic clinical trials have proven the efficacy and safety of monoclonal antibodies against PD-1 and PD-L1 in the treatment of advanced NSCLC patients. The Keynote-021 study included 123 NSCLC patients with stage IIIB to IV non-squamous metastases. Among them, 60 patients received pembrolizumab combined with chemotherapy and 63 patients received chemotherapy alone.^[10]^ The results showed that the curative effect in the combination therapy group was markedly better than that in the chemotherapy alone group, with the difference reaching statistical significance. The Keynote-042 study further demonstrated that pembrolizumab could achieve a relatively positive curative effect in NSCLC patients with negative expression of PD-L1, and that its efficacy was enhanced with increasing PD-L1 expression.^[11]^ Meanwhile, the CheckMate-017, CheckMate-057, OAK, and other stage III clinical trials investigated PD-1 inhibitors (nivolumab, pembrolizumab) and PD-L1 inhibitors (atezolizumab, durvalumab) as second-line therapeutic regimens after the failure of first-line treatment with platinum-based chemotherapy.^[12,13]^ The results clearly indicated that PD-1 and PD-L1 inhibitors could lengthen the overall survival time and median survival time in comparison with docetaxel regimens. Consequently, the Food and Drug Administration approved the application of pembrolizumab, nivolumab, atezolizumab, and durvalumab as second-line treatments for advanced NSCLC patients.^[14]^ In China, investigation and application of immune checkpoint inhibitors in advanced NSCLC patients remain in the start-up stage, and there are no reliable data and clinical trials to support their use.

Immune checkpoint inhibitors can provide synergistic effects with radiotherapy. In the Keynote-001 study involving 98 advanced NSCLC patients, progression-free survival and overall survival in the radiotherapy plus pembrolizumab combination group were 4.4 months and 10.7 months, respectively, being superior to the corresponding values of 2.1 and 5.3 months in the pembrolizumab alone group (*P* < .05), with tolerance to therapy-related toxicity.^[15]^ The Pembro-RT study was a prospective, multicenter, randomly-assigned stage II clinical trial involving 78 advanced NSCLC patients who were divided into a pembrolizumab plus radiotherapy combination group and a pembrolizumab alone group. The results demonstrated that in the 12th week, the objective remission rates in the 2 groups were 36% and 18%, respectively, with no significant difference in terms of therapy-related toxicity.^[16]^ In the trial, external beam radiation therapy was performed on metastatic tumors in the right ilium, basis cranii, and cervical vertebra during the immune checkpoint inhibitor therapy. After the radiotherapy, the in-field lesions had essentially disappeared, while the sizes of out-of-field left ilium metastatic tumors and left posterior chest wall neoplasms were markedly reduced and replaced by normal bone tissues, indicating remote effects of the radiotherapy. Nevertheless, this represents a valuable but rare circumstance. At the present time, researchers commonly believe that the orthotopic treatment effect with radiotherapy is related to a reconstruction effect of the immune microenvironment, but the potential mechanism remains elusive.^[17,18]^ Some researchers have also pointed out the safety issues for radiotherapy combined with immunotherapy and suggested that radiotherapy will probably cause harm to immune cells within the irradiated volume and lead to myelosuppression. Thus, further studies are warranted to explore suitable radiotherapy segmentation methods and choices of immune checkpoint inhibitors toward future applications.

In conclusion, in the present case, the authors chose the combination chemotherapy of paclitaxel liposomes and nedaplatin, and the curative effect evaluation after the second cycle of chemotherapy was progression of disease. On the basis of the above-mentioned theoretical foundations and research reports and after considering the unique features of the patient, the authors selected a PD-1 inhibitor as the second-line treatment after the failure of the first-line chemotherapy. During the treatment process, external beam radiation therapy was conducted on local bone metastases (cervical vertebra and lium), which markedly alleviated the disease and produced a constant effect. At the present time, the treatment mode of combining immune checkpoint inhibitors and chemotherapy for advanced NSCLC patients remains at an initial stage with limited studies and reports. This article is designed to provide a clinical basis for the best possible therapeutic strategies for advanced NSCLC patients through a case report.

## Author contributions

**Conceptualization:** Yonglong Jin, Yinshi Cui, Bo Li, Xiguang Liu.

**Formal analysis:** Yonglong Jin.

**Funding acquisition:** Yonglong Jin.

**Investigation:** Wenjing Xiao, Xintong Wang, Yinshi Cui, Bo Li, Xiguang Liu.

**Methodology:** Wenjing Xiao, Xiguang Liu.

**Visualization:** Xiguang Liu.

**Writing – original draft:** Yonglong Jin.

**Writing – review & editing:** Xiguang Liu.
